# Osteopoikilosis Demonstrating Multiple Joint Involvement in an Adult Male: An Incidental Radiographic Finding

**DOI:** 10.7759/cureus.3253

**Published:** 2018-09-04

**Authors:** Ariel Botwin, Christopher Wasyliw

**Affiliations:** 1 University of Central Florida College of Medicine, Orlando, USA; 2 Diagnostic Radiology, Florida Hospital-Orlando, Orlando, USA

**Keywords:** osteopoikilosis, sclerotic lesion, radiography, incidental finding

## Abstract

Osteopoikilosis is a rare condition that is characterized by multiple small non-aggressive appearing sclerotic foci in a periarticular distribution. Typically, it does not cause any symptoms and is diagnosed incidentally on imaging studies done for other reasons. We present a case of osteopoikilosis in a 37-year-old male, which was diagnosed incidentally on radiographs.

## Introduction

Osteopoikilosis is a benign sclerosing bony dysplasia that can occur sporadically or be inherited in an autosomal dominant fashion [1]. Its incidence is estimated to be one in every 50,000 individuals [2, 3]. On radiographs, it is characterized by multiple sclerotic foci that correspond to condensations of the lamellar trabeculae on histology [2]. Roughly 15–20% of patients may present with joint pain and effusion but the majority of patients are asymptomatic and diagnosed incidentally on imaging studies performed for an unrelated clinical indication [3].

## Case presentation

**Figure 1:**
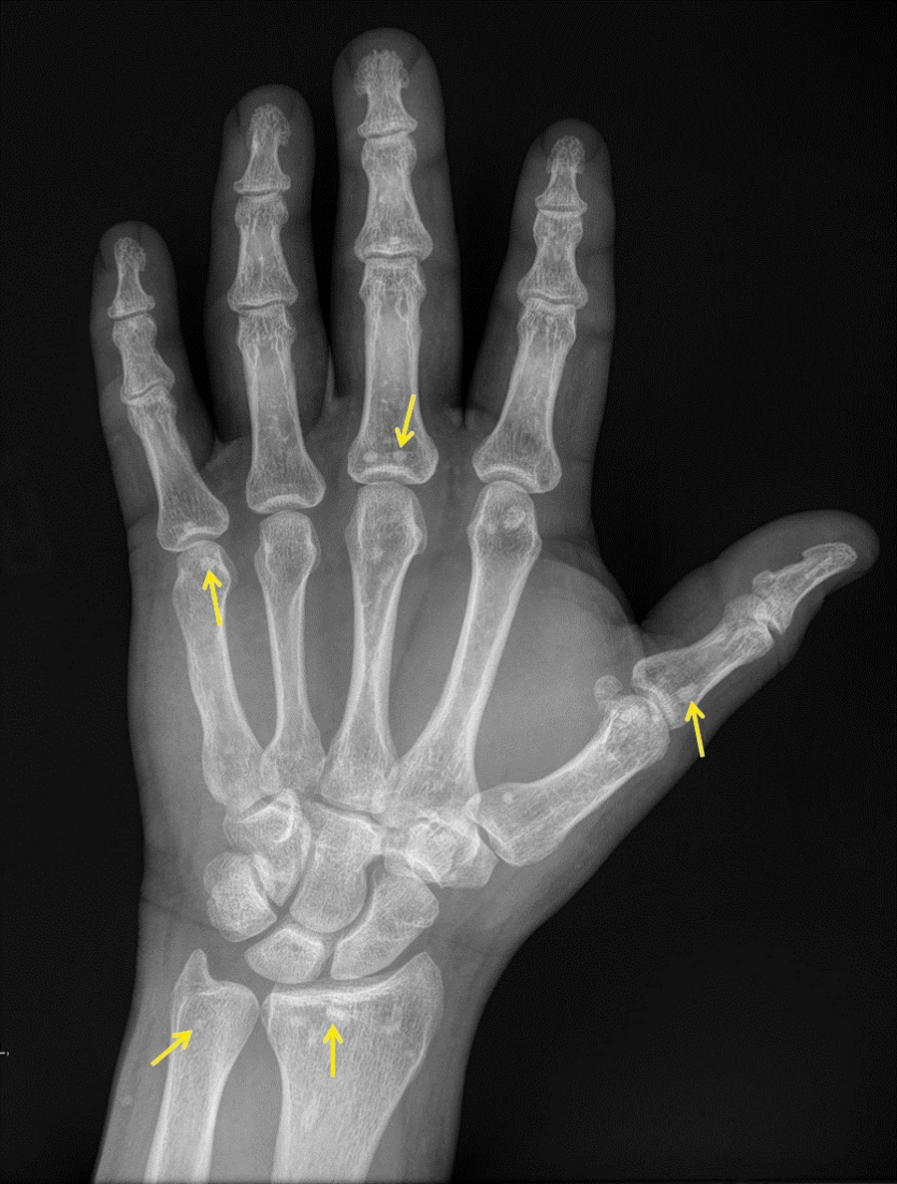
Anteroposterior Radiograph of the Left Hand. Multiple small, non-aggressive, periarticular sclerotic foci (yellow arrows) are visualized at the distal radius, distal ulna, first carpometacarpal joint, first through fifth metacarpal phalangeal joints, second and third proximal interphalangeal joints, and fifth distal interphalangeal joint.

A 37-year-old male patient presented to the emergency department with a puncture wound to the left hand that he had sustained while working with machinery. His medical history was negative for any cancer or chronic musculoskeletal complaints such as joint pain, weakness, or limited range of motion. Physical exam revealed no weakness, loss of range of motion, or numbness of the affected digits. A radiograph of the left hand demonstrated no fracture, dislocation, or foreign body. However, multiple small non-aggressive appearing periarticular sclerotic foci were visualized incidentally (Figure 1).

**Figure 2:**
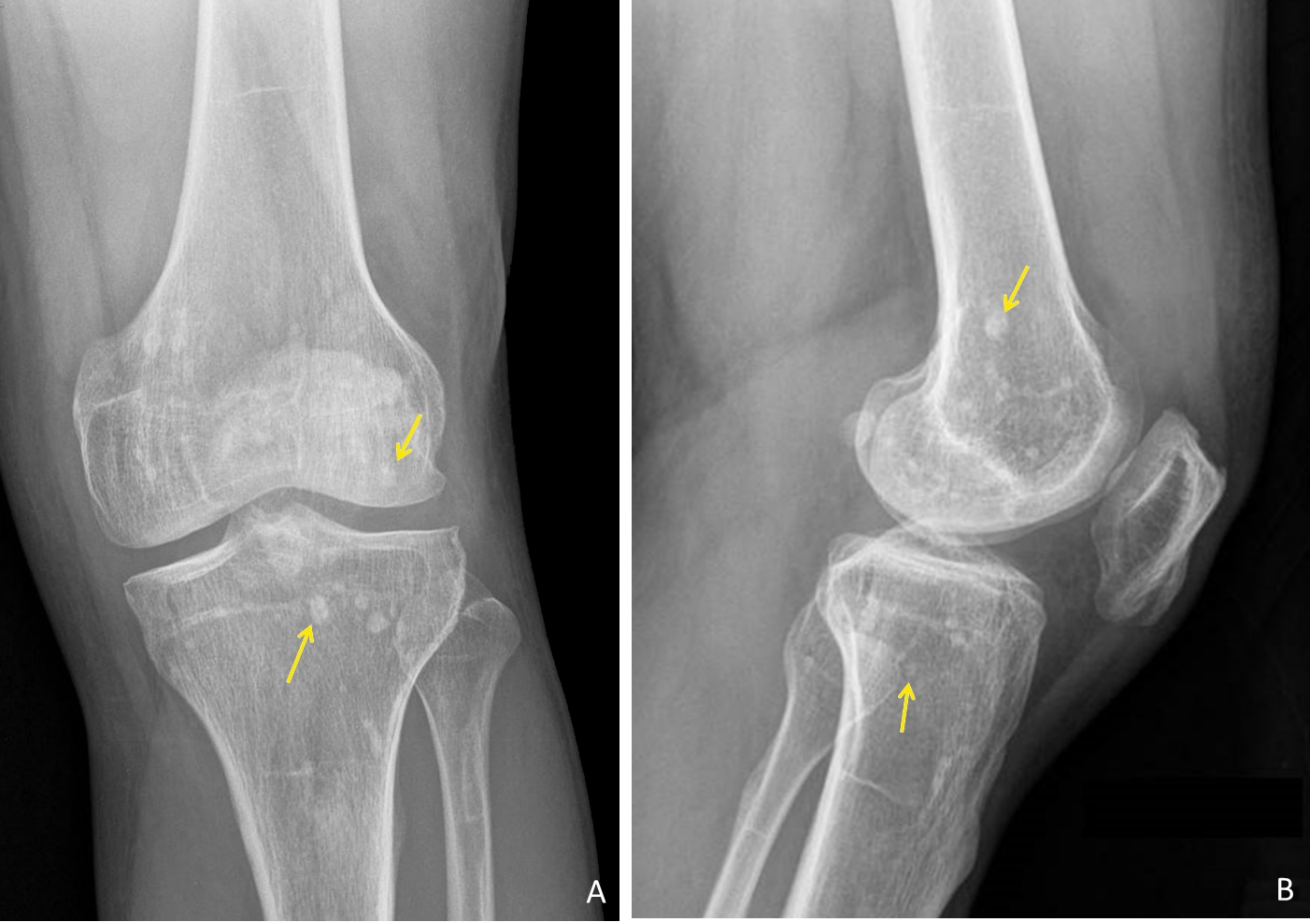
Radiographs of the Left Knee. Anteroposterior (A) and lateral (B) radiographs of the left knee demonstrating multiple non-aggressive appearing, sclerotic foci (yellow arrows) in the distal femoral and proximal tibial metaphysis and epiphysis, as well as in the proximal fibular metaphysis.

**Figure 3:**
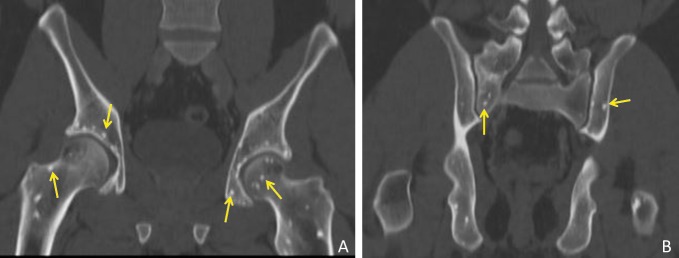
Coronal Reformatted Computed Tomography (CT) of the Pelvis. Small non-aggressive appearing sclerotic foci (yellow arrows) are visualized involving the periarticular (A) right and left hip and (B) sacroiliac joints.

**Figure 4:**
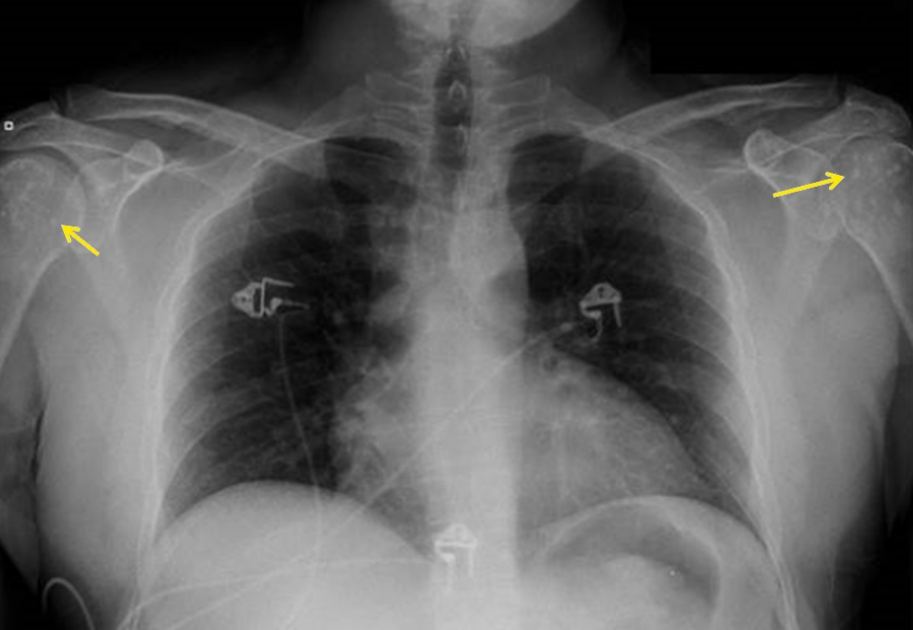
Posteroanterior Radiograph of the Chest. Non-aggressive periarticular sclerotic lesions (yellow arrows) are visualized at the bilateral glenohumeral joints.

Further review of the patient’s prior imaging studies revealed that similar appearing periarticular sclerotic foci were present in other areas as well, including the left knee (right not imaged), bilateral shoulders, hips and sacroiliac joints (Figures 2-4). The radiographic findings coupled with the patient’s medical history are compatible with the diagnosis of osteopoikilosis.

## Discussion

Osteopoikilosis classically appears on radiographs and computed tomography (CT) as multiple, small, well-defined radiodense foci of cortical-like bone in the medullary space parallel to the trabeculae typically involving the carpals, tarsals, pelvis, and epiphyseal and metaphyseal ends of the long bones in a symmetric distribution [4, 5]. The axial skeleton, with the exception of the sacrum, is typically uninvolved. They develop during childhood equally in males and females and do not regress, thus are found in all ages. The foci are similar in size usually ranging from 2 to 10 millimeters [3]. The patient in our case has periarticular involvement, which is the typical distribution described in the literature. His sacroiliac and hip joints are symmetrically involved bilaterally, while the rest of the pelvis is spared. In his hand, there is involvement at the radiocarpal, ulnocarpal and metacarpal phalangeal joints as well as several interphalangeal joints. Additionally, there is involvement at the bilateral glenohumeral joints and left knee. In a study of 53 subjects with osteopoikilosis, the humerus and tibia were affected in only 28.2% and 20.5% of subjects, respectively [6]. On magnetic resonance imaging, the lesions have low signal intensity on both T1- and T2-weighted sequences [3]. Additionally, the lesions typically display no activity on scintigraphy [2].

Occasionally, osteopoikilosis may mimic that of other conditions, such as sclerotic metastases, mastocytosis, and tuberous sclerosis, which all require further medical attention [7]. In contrast to patients with sclerotic metastases, patients with mastocytosis or tuberous sclerosis typically have a clinical presentation highly suggestive of these conditions by the time bone lesions can be identified on radiograph [1]. Also, the sclerotic lesions of mastocytosis are usually more scattered rather than periarticular and involve the axial skeleton [8]. As for tuberous sclerosis, the lesions can be periarticular near the sacroiliac joints, but typically have a mottled appearance [9]. Sclerotic metastases may display axial skeleton involvement, osseous destruction, varying sizes, and asymmetric distribution [7]. However, if these features and the classic appearance of osteopoikilosis are not present, the two conditions may be difficult to distinguish on radiography alone. In fact, a retrospective study reported that five of 10 patients with osteopoikilosis who presented to an orthopedic department were mistakenly diagnosed with osteoblastic metastases initially [10]. Failure to reach an accurate and timely diagnosis can expose patients to unnecessary diagnostic tests and complications. Given the classic imaging features with our case as described above, the diagnosis of osteopoikilosis can be confidently made.

## Conclusions

Osteopoikilosis is a rare condition that is usually diagnosed on radiographic studies incidentally. In cases where the classical imaging characteristics of osteopoikilosis are not present, these benign lesions can be mistaken for sclerotic metastases or other more serious conditions. In our case, the patient demonstrated the pathognomonic radiographic appearance of osteopoikilosis, which is an important finding for radiologists and clinicians to recognize.
